# Rewriting the Treatment Paradigm: Ilizarov Method Achieves High Success in Septic Non-Unions Without Local Antibiotics or Biologic Adjuncts

**DOI:** 10.3390/biomedicines13071665

**Published:** 2025-07-08

**Authors:** Filippo Vandenbulcke, Andrea Dorotei, Emiliano Malagoli, Alexander Kirienko

**Affiliations:** 1 Department of Biomedical Sciences, Humanitas University, Via Rita Levi Montalcini 4, 20072 Pieve Emanuele, Milan, Italy; emiliano.malagoli@humanitas.it (E.M.); alexander.kirienko@humanitas.it (A.K.); 2 IRCCS Humanitas Research Hospital, Via Manzoni 56, 20089 Rozzano, Milan, Italy; 3 IRCCS Policlinico San Donato, Piazza Edmondo Malan 2, 20097 San Donato Milanese, Milan, Italy; andrea.dorotei@gmail.com

**Keywords:** infected non-union, infected bone defect, Ilizarov Distraction Osteogenesis, non-union, Ilizarov method, external fixation

## Abstract

**Background/Objectives**: The aim of this study is to describe the characteristics of a cohort of patients who underwent surgery for septic non-union of the lower extremities. **Methods**: We analyzed clinical data from 74 patients affected by septic non-union of long bones in the lower extremities, treated with the Ilizarov method between January 2006 and December 2021. The primary objective of our study was to describe the time from surgery to bone union. **Results**: Patients had undergone a median of three previous surgical interventions, had an average bone defect of 5.4 cm, with 43.4% of patients having a Non-Union Scoring System (NUSS) > 75 points, and 46.5% of patients having been considered candidates for limb amputation in other centers. Bone union was achieved in 73 patients (98.65%), while infection resolution was achieved in 68 patients (91.89%). In 63 patients (85.13%), healing was obtained with one surgical procedure only. Only 11 re-interventions were necessary after frame removal (14.86%): 10 were due to re-fractures (13.51%) and 1 to an infection recurrence, which resulted in an amputation (1.35%). At a time of 6.01 ± 3.9 years follow-up, the Association for the Study and Application of the Methods of Ilizarov (ASAMI) scoring system indicated excellent or good outcomes in 97.3% for the bone subscale and in 89.2% for the functional subscale. The Patient Global Impression of Change (PGIC) showed that 96.8% of patients were “very much improved” or “much improved”. Patients who have suffered a more recent trauma or fewer previous surgeries achieved a better outcome. **Conclusions**: Despite some limitations, this study shows that treatment of septic non-unions using the Ilizarov method is both highly effective in bone and infection healing and results in a satisfactory functional outcome. The results observed in our cohort suggest that the Ilizarov method could be critically re-evaluated as a primary treatment option for these challenging cases. The clinical relevance of these findings lies in their potential to significantly alter the current treatment paradigm, by questioning the need for biologic adjuncts and local antibiotics, thereby reducing healthcare costs.

## 1. Introduction

Infected non-union of long bones with substantial bone defects represent a challenging clinical scenario that is often very difficult to treat and even more difficult to cure permanently [1]. A long bone fracture is typically classified as a non-union when it fails to heal without additional surgery [2]. According to the U.S. FDA definition, a fracture can be classified as a non-union nine months after injury or when there is a failure of progression towards union over the previous three months [3,4]. However, a specific duration of time to define non-union must be related to the location of the fracture and the severity of the original injury [5]. Long bone non-unions are frequently encountered in clinical practice following high-energy trauma and are notoriously difficult to manage [6,7,8]. The presence of infections and devascularization within large bone defects prolong treatment duration, worsen prognosis, and, in some cases, may lead to lower limb amputation [1]. Significant patient-related risk factors for the development or the persistence of non-union have been identified, such as smoking and diabetes mellitus [9]. Additional critical challenges include intercalary bone loss, whether from the initial injury or from subsequent surgical debridement, deformities, leg-length discrepancies (LLDs), failure of internal fixation devices, joint stiffness, and polymicrobial infections caused by resistant pathogens [10,11]. A thorough understanding of the etiological agents is crucial for effective management. While a broad spectrum of microorganisms can cause infection in non-union sites, common pathogens frequently isolated include Staphylococci, and Enterococci [1,12]. These bacteria, often forming biofilms, contribute significantly to the persistence of infection and impede the natural healing process, necessitating comprehensive strategies that address both the infection and the non-union simultaneously [1]. The complex and costly management of this condition often results in psychological, social, and economic challenges [13]. The primary objectives of treatment are the eradication of infection, the reconstruction of the bone, and the achievement of bony union [2,14]. Several techniques have been developed to achieve these goals [15].

Surgical management has evolved through bone transport techniques, originally developed by G. Ilizarov, that utilize distraction osteogenesis (DO) to bridge large bone gaps [16]. The great advantage of this technique is its ability to fully eradicate infection by entirely removing the avascular and potentially infected bone segment and to create new bone through bone transport. It also fills the intercalary gap while simultaneously correcting LLDs and axial deformities [6,17,18,19,20,21,22,23,24,25,26,27]. Union rates up to 97% have been reported, even for large bone defects exceeding 3 cm [26]. However, some studies have shown some complications such as pin-tract infections, axial deviation, stiffness in adjacent joints, and wire loosening [28,29]. Other techniques, such as the Masquelet Induced Membrane Technique (IMT), vascularized fibular grafts, new biomaterials or bone substitutes, can be performed as either a one-stage or two-stage procedure [30,31,32].

Current treatment paradigms for septic non-unions typically advocate for a multi-faceted approach combining radical surgical debridement, stable mechanical fixation, and often, the concomitant use of local antibiotic delivery systems or various biologic adjuncts to enhance bone healing and control infection [2,33]. However, the conclusive evidence supporting the routine application of these adjuncts, particularly in the context of active infection where their efficacy may be compromised and resistance concerns are paramount, remains limited [25,34,35,36,37,38]. We hypothesized that the Ilizarov method, even without routine local antibiotics or biologic adjuncts, could achieve high rates of bone union and infection resolution in septic non-unions.

## 2. Materials and Methods

This is a monocentric observational retrospective cohort study conducted in our institution in agreement with the Good Clinical Practice, the current version of the Declaration of Helsinki, and the applicable regulations. The Local Ethics Committee has approved the study (23 April 2024—n.21/24).

Medical records from January 2006 to December 2021 were reviewed to collect clinical information about patients undergoing treatment with the Ilizarov method for septic non-union.

Subjects fulfilling all of the following criteria were included:

- adult patients (>18 years);
- diagnosis of infected non-union of long bones of the lower extremity according to the Fracture-Related Infection (FRI) consensus definition by the European Bone and Joint Infection Society (EBJIS) [39];
- treatment with the Ilizarov method at our institution.

The presence of any one of the following criteria led to the exclusion of the subject:

- known inflammatory systemic diseases at the time of enrolment;
- patients who have undergone any bone transport other than longitudinal;
- patients who did not consent for the use of their data in clinical research at the time of admission.

### 2.1. Study Objectives

The objective of our study was to describe the characteristics of a cohort of patients who were operated on in our institution for infected non-union with the Ilizarov method. The primary objective of our study was to describe the time from surgery to bone union, as shown on X-rays and confirmed by mechanical test before frame removal [4,40]. For this purpose we chose, as a primary end-point, bone union survival, expressed as months from the first surgical intervention to the last frame removal after eventual re-intervention and as the number of revisions needed to obtain healing. The secondary objective was to describe the outcomes and the complications of the intervention.

Secondary end-points were the following: blood transfusion rate; re-intervention free survival; amputation rate; infection healing rate (defined according to the FRI consensus definition by the EBJIS [39]); External Fixation Time (EFT), which indicates the total duration of external fixator application; External Fixation Index (EFI), that is calculated by dividing the months of external fixation by the cm of bone lengthening and is an index of treatment efficiency; rate of patients walking without aids; return to work rate; Patient Global Impression of Change (PGIC), assessing the overall patient-reported change; Association for the Study and Application of the Methods of Ilizarov (ASAMI) score; EQ-5D-5L; and LEFS (Lower Extremity Functional Scale).

### 2.2. Statistical Analysis

The study included all the suitable patients operated in a 16-year period (from January 2006 to December 2021) with at least a 2-year follow-up. Since the primary outcome was purely descriptive, no a priori sample calculation could be performed. The final sample size allowed error estimates under 6%.

Data are described as frequencies and percentages for qualitative variables, as means and standard deviations (SD) for approximately Gaussian quantitative variables, or as medians, minimum and maximum value (range), and first and third quartiles (interquartile range). Adherence to Gaussian distribution was evaluated with the Shapiro–Wilk test.

Regarding the primary end-point, we considered a patient as healed if his course of treatment had ended. The time to heal was considered as the time between the date of first surgery and the date of frame removal. For non-healed patients the last contact date was considered. Amputated patients were considered as censored, and the amputation date was considered as the censoring date. The same analysis was also conducted, using the number of revisions necessary to reach healing as survival time. Re-intervention free survival was calculated only for healed patients. The time was calculated from the date of frame removal to the re-intervention date. A patient was considered censored if no re-intervention occurred. All survival curves were calculated using the Kaplan–Meier method, and data were expressed as survival median. Changes in variables during visits were explored using multilevel mixed-effects linear regression. All statistical tests were performed using a two-sided approach and were considered significant when *p* value was inferior to 0.05. All analyses were performed with Stata version 18.

## 3. Results

### 3.1. Patient Characteristics

**Figure 1:**
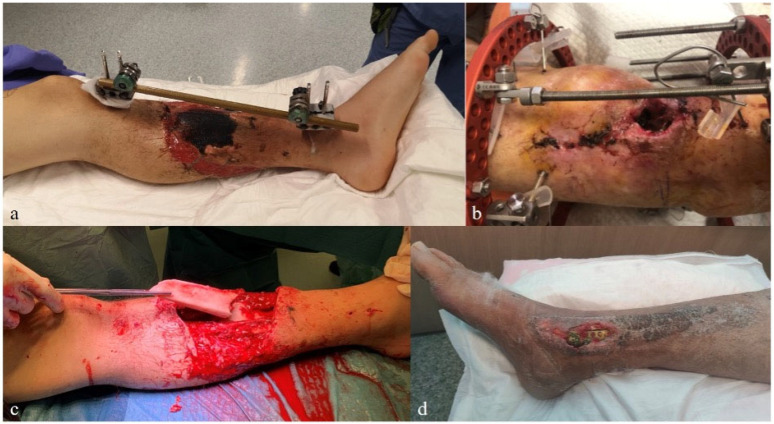
Pictures of patients included in the study presenting infected non-union associated to massive area of soft tissue (**a**) and bone (**c**) necrosis, soft tissue defects and exposure of hardware (**b**,**d**).

We included 74 patients operated by the senior author (AK). The average patients’ age at surgery was 43.0 ± 14.4 years. A total of 56 patients were male (75.7%). The affected side was right in 34 cases (45.95%). The average Non-Union Scoring System (NUSS) score [41] was 69.9 ± 8.6 points; 24 patients (32.4%) scored a NUSS greater than 75 points. Patients had undergone a median of three previous surgical procedures (range 1–32). The median time from injury to our intervention was 14.3 months (range 0.7–298.1). Amputation had been proposed in other institutions to 33 patients (46.5%). The affected site was the distal metaphyseal tibia in 29 cases (39.2%), the diaphyseal tibia in 22 cases (29.7%), the distal metaphyseal femur in 11 cases (14.9%), the proximal metaphyseal tibia in seven cases (9.5%), and the diaphyseal femur in five cases (6.8%). See Table 1. A few clinical pictures are shown in Figure 1.

### 3.2. Treatment Description

**Figure 2:**
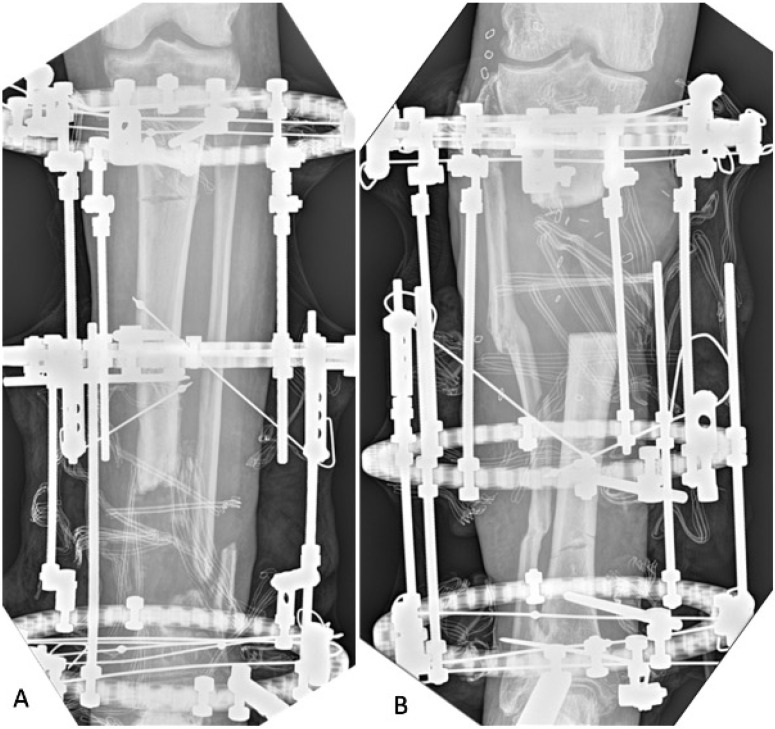
X-rays of two different patients treated by resection of the non-union site and anterograde (**A**) or retrograde (**B**) bone transport.

During surgery previous implanted hardware was removed. This consisted of 17 plates and screws (22.97%), 13 external fixators (17.56%), 12 intramedullary nails (16.21%), 3 screws (4.05%), and 1 cement spacer (1.89%). No hardware was removed in 27 cases (36.48%). An accurate pre-operative planning of the non-union site resection was performed based on X-rays, Computerized Tomography (CT) and Magnetic Resonance Imaging (MRI) scan, and 2-deoxy-2-[18F]fluoro-D-glucose positron emission tomography-computed tomography ([18F]FDG PET/CT). The planned resection was confirmed intra-operatively by macroscopic signs of bone viability, such as the “paprika sign”. According to the defect size, local soft tissue conditions and patient characteristics, one of the following treatment strategies has been chosen. For smaller defects, less than approximately 3 cm [42], our preferred option was one-stage open resection, acute shortening and osteotomy for re-lengthening (32 cases—43.24%). To address longer defects, after open resection, bone transport (BT) was performed in 24 cases (32.4%), respectively, as a one-stage procedure in 14 cases (18.91%) that did not need a docking site revision, and as a two stages in 10 cases (13.51%) requiring a docking site revision. To avoid overburdening either the local limb condition or the patient’s general condition, an open resection, acute shortening, followed by a second stage osteotomy for re-lengthening was performed in ten cases (13.51%). In eight patients (10.81%) minimal debridement was required and no reconstruction additional procedures were indicated, so the treatment strategy included open resection of the non-union site and fixation. See Figure 2.

The average bone defect was 5.4 ± 2.7 cm including previous shortenings and the defect caused by surgical resection. Surgical time was 196.1 ± 43.4 min. In cases of two-stage treatment strategy, the duration from the first to second stage surgical procedure was 4.7 ± 3.1 months. The median post-operative hospitalization duration was 4 days (range 1–21) and only one patient needed blood transfusion during post-operative course. Multiple tissue specimens (≥3) were taken intra-operatively, each with clean instruments. The culture exam results were monomicrobial in 38 patients, polymicrobial in 15 patients, negative in 17 patients, and not available in 4 patients. Among the main bacteria detected, Staphylococcus Aureus (24%), Staphylococcus Epidermidis (24%), and Enterococcus Faecalis (11%) were the most common. Isolated pathogens are detailed in Table 2.

### 3.3. Outcomes

**Figure 3:**
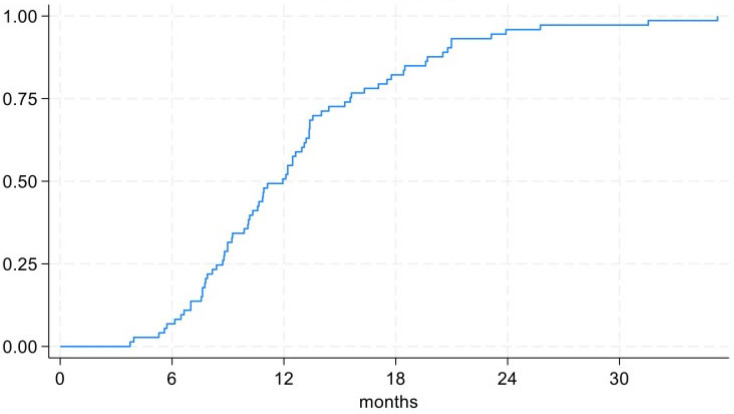
Bone union curve.

**Figure 4:**
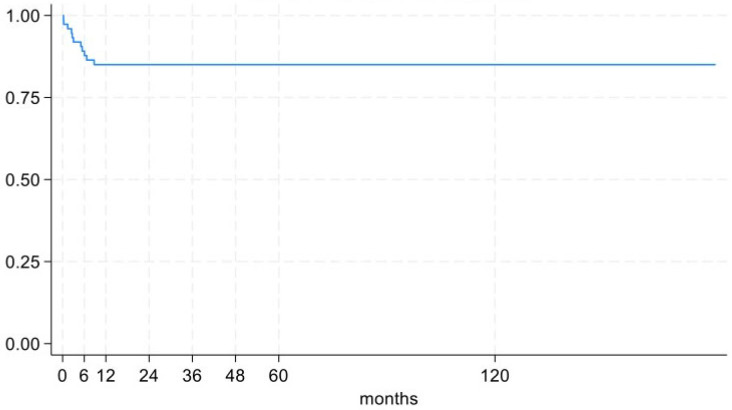
Re-intervention free survival curve.

Bone union was achieved in 73 patients (98.65%, 95%CI 92.70% to 99.97%) at an average time of 12.8 ± 6.0 months, while the infection healed in 68 patients (91.89%, 95%CI 83.18% to 96.97%). The clinical presentation of patients who achieved bone union but not complete infection resolution was consistent with a controlled chronic osteomyelitis. Survival curves are shown in Figure 3 and Figure 4. The external fixation time (EFT) was 11.5 ± 4.4 months. The external fixation index (EFI) was 2.49 ± 1.09.

**Figure 5:**
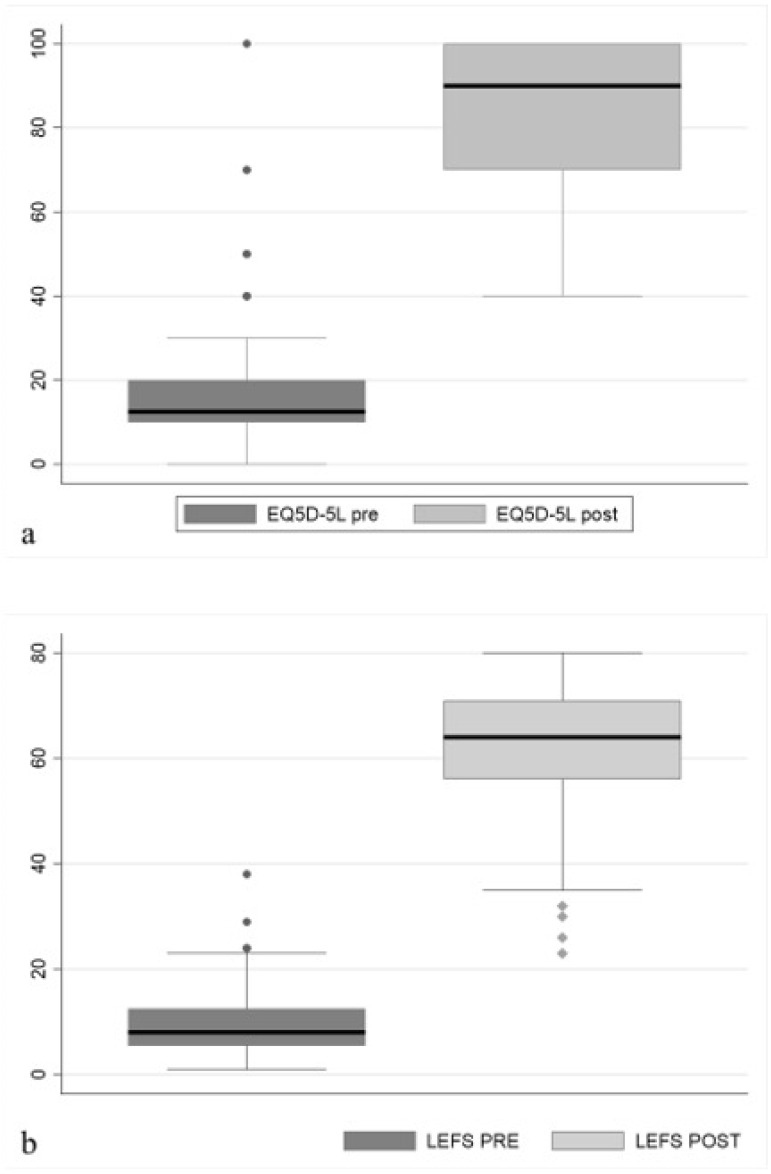
Box plot demonstrating the LEFS (**a**) and EQ-VAS (**b**) increase after surgery (*p* < 0.05).

Concerning clinical assessment, EQ VAS increased by a pre-operative median of 12.5 points (range 0–70) to a post-operative median of 90 (range 40–100) at time of last follow-up, improving by 77.5 points (*p* < 0.05). LEFS increased by 53 points (range 3–79) passing from a pre-operative median of 8 (range 1–38) to a post-operative median of 64 (range 23–80) at last follow-up, improving by 630% (*p* < 0.05). See Figure 5. A higher post-operative LEFS was associated with a shorter time from injury to our intervention by a coefficient of −0.21 (CI: 0.40–0.02; *p* < 0.05).

Excellent or good ASAMI scoring system was achieved in 97.3% for the bone subscale and in 89.2% for the functional subscale. Patients achieving an excellent functional outcome underwent a median of two previous surgical procedures (2.0; range 1–9), which was significantly lower (*p* < 0.05) compared to those with a good functional outcome (3.5; range 1–32). No other associations were found between functional results and pre-operative characteristics, meaning that there were no statistically significant associations between clinical outcomes and the size of bone defect, the NUSS, the previous indication to amputation.

What is more, 92% of patients were able to walk without aids, only 23% used a crutch for long distances and 4% used a wheelchair for long distances. A total of 93% of patients went back to work to do the same task as prior to surgery (77%) or were reassigned (16%). Patient Global Impression of Change (PGIC) showed that very much improved was 89% and much improved was 7.8%. Finally, 96.9% of patients declared they would repeat the same treatment. The mean follow-up time was 6.01 ± 3.9 years. See Table 3.

### 3.4. Unplanned Revisions and Re-Interventions

A total of 63 patients (85.13%) healed without further surgery. One unplanned surgical revision was needed in ten cases (13.51%) at a median time of 7.44 ± 2.11 months from the intervention. Five patients (6.75%) were revised for delayed union (three underwent open revision of the non-union site and two underwent closed osteotomy with chipping technique); three patients (4.1%) underwent frame renewal for half-pin and wire loosening; two patients (2.7%) underwent surgical debridement for infection persistence. Three cases (4.1%) required a second unplanned surgical revision for other reasons than infection or non-union, such as correction of residual deformities or early consolidation of the osteotomy. There were 11 re-interventions after frame removal (14.86%): 10 re-fractures (13.51%) underwent new fixation with Ilizarov apparatus associated to closed osteotomy with chipping technique in 5 cases, non-union site resection, ASR, in three cases, and open revision of the non-union site in 2 cases; 1 infection recurrence underwent amputation (1.35%) in a patient with bilateral infected non-union of the femur. See Table 2.

## 4. Discussion

Septic non-unions represent a significant clinical problem with multifaceted implications, including substantial economic burden and significant psychosocial implications for patients, including persistent pain, reduced quality of life, and potential disability [13,43]. Moreover, the economic impact of fracture non-unions is considerable, affecting both individuals and healthcare systems. Infection and non-union after long-bone fractures often require prolonged medical attention, multiple surgical interventions, and extensive rehabilitation and are associated with large increases in costs compared to an uncomplicated fracture: from 150% to 800% in case of infection, and from 260% to 430% in case of non-union [43].

As stated by Calori and co-workers, a NUSS score from 76 to 100 points may indicate the need for primary amputation, arthrodesis, prosthesis or mega-prosthesis implantation depending on the patient’s condition, the severity of bone loss, and anatomical localization [41]. In the present study, the average NUSS was 69.9 ± 8.6, and 32.4% of patients had a NUSS greater than 75, a value for which limb amputation is recommended. In fact, amputation had been proposed in other institutions to 46.5% of the patients. Following our intervention, only one patient was amputated. What is more, candidates for amputation had a satisfactory functional outcome with no statistically significant differences compared with other patients. These results should induce us to reconsider the real indications for amputation in orthopedics. Furthermore, shorter time from injury to our intervention and a lower number of previous surgical procedures were associated with a better functional outcome. All this suggests that this treatment should not be considered as a last resort only.

Moreover, deep intra-operative tissue samples were culture-negative in 28% of patients. Although such results may seem counterintuitive, this percentage is comparable to the culture-negative rate in periprosthetic joint infections (PJI) [44]. There are two schools of thought in the treatment of infected non-union: the ‘infection-elimination first’ strategy and the ‘union-first’ strategy [25]. The former strategy aims at eliminating the infection as the first and major objective of bone union is the second objective. Conversely, the authors of this paper follow the second strategy which aims at achieving union first and then dealing with the problem of infection if it arises [25]. Indeed, no routine intravenous empirical antibiotic therapy was administered post-operatively, as is common practice in the treatment of infected non-unions, which typically requires long-term hospitalization. In this study, the average hospital stay was only four days, and in recent years we further shortened it to two days, which also reduced costs related to hospitalization. After early discharge, targeted antibiotic therapy was prescribed in specific cases, such as those with signs of local infection or when particularly aggressive pathogens had been isolated.

While the Ilizarov method’s efficacy in treating complex non-unions is well-documented [6,26,27], a distinguishing feature of our series is the deliberate omission of routine local antibiotic delivery systems and biologic adjuncts. Many published reports on septic non-unions frequently describe the adjunctive use of antibiotic carriers, or various bone substitutes and growth factors [2,33]. Our favorable outcomes suggest that meticulous surgical debridement, combined with the comprehensive mechanical stability and bone regeneration capabilities of the Ilizarov apparatus, can be highly effective without the routine need for these surgical augmentations. This approach potentially offers advantages by simplifying treatment protocols, reducing direct and indirect costs associated with material usage and prolonged hospital stays, and mitigating concerns regarding antibiotic resistance development or complications related to foreign body implantation. Our findings therefore contribute to a critical re-evaluation of what constitutes the ‘essential’ components of care in septic non-unions, suggesting that the primary focus should remain on aggressive debridement and robust mechanical stabilization.

For the patients who achieved bone union but not complete infection resolution, their clinical presentation was consistent with a controlled chronic osteomyelitis, which, despite its persistent nature, allowed for satisfactory limb function and good functional recovery, leading these patients to decline further surgical interventions focused solely on infection eradication due to the acceptable quality of life achieved.

Interestingly, all re-interventions were performed within the first nine months after frame removal; thus, following this period, no patient underwent further surgery, with a 6.01 years average follow-up. Therefore, treatment of septic non-unions using the Ilizarov method can be considered a definitive intervention. In addition to being effective and definitive, the use of the Ilizarov method has limited costs. Indeed, in 73% of cases, a single surgery was sufficient for healing, despite these patients undergoing a median of three prior surgeries. The surgical approach is minimally invasive with limited blood loss, as only 1.3% of cases required a blood transfusion post-operatively. Moreover, infected non-union treatment, following the Ilizarov philosophy, does not require the use of local antibiotics or biologic treatments. In fact, during our study, no scaffolds, bone substitutes, allografts, stem cells, or growth factor augmentations were used, which significantly reduced treatment costs. Orthobiologics are widespread in everyday practice, although there is little evidence for their current use when treating non-unions [45]. What is more, the effects of mixing antibiotics with bone grafting (in terms of bone union or incorporation) are not known [25,34,35,36,37,38]. Additionally, the classical Ilizarov frame with rods and hinges was used in all patients, and no hexapod system was employed, which would have significantly increased treatment costs. The fact that bone and infection healing was achieved without these augmentations further emphasizes the primary importance of stability in treating this condition, which is difficult to achieve with other fixation hardware.

This study, while providing valuable insights into the management of septic non-unions with the Ilizarov method, is not without limitations. Firstly, its retrospective, single-center design inherently introduces potential for selection bias and limits the generalizability of our findings to other populations or clinical settings. The absence of a comparative control group precludes definitive conclusions regarding the superiority of the Ilizarov method over alternative treatment modalities for septic non-unions. While our cohort of 74 patients represents one of the largest single-center series on this complex condition, the inherent heterogeneity of septic non-unions, characterized by varying bone defects, infection severities, and patient comorbidities, presents a challenge for standardization and comparative analysis. Furthermore, while we provide long-term follow-up data, some very late complications or subtle functional impairment may not have been fully captured. Lastly, although validated outcome measures were utilized, some are patient-reported, introducing a degree of subjectivity. Future prospective, multi-center studies with comparative groups would be beneficial to further validate these findings and strengthen the evidence base.

## 5. Conclusions

In conclusion, this retrospective study suggests promising efficacy of the Ilizarov method in achieving high rates of bone union and infection resolution within our specific cohort of patients with complex septic non-unions of the lower extremities, even without the routine use of local antibiotics or biologic adjuncts. The favorable success rates and functional outcomes observed in this series indicate that the Ilizarov method warrants further consideration as a highly effective and viable treatment option for these challenging cases.

Furthermore, these results raise important questions regarding the routine indications for local antibiotics and orthobiologic techniques for surgical augmentation, which are often considered essential adjuncts in the treatment of infected non-union. This work highlights the potential for streamlining treatment protocols by minimizing the need for additional interventions and agents, thereby potentially reducing healthcare costs.

The clinical relevance of these findings lies in their potential to contribute to refining the current treatment paradigm, by offering a robust strategy that may reduce the risk of amputation and aid in restoring function in a patient population often facing dire prognoses. However, given the observational nature and inherent limitations of this study, further prospective, comparative studies are warranted to definitively validate these findings and their broader applicability.

## Figures and Tables

**Table 1:** Patient characteristics.

Characteristics	Value
**Age at surgery—years**	
Average ± SD	43.0 ± 14.4
**Sex—no (%)**	
Male	56 (75.7%)
**Affected site—no (%)**	
Diaphyseal femur	5 (6.8%)
Distal metaphyseal femur	11 (14.9%)
Proximal metaphyseal tibia	7 (9.5%)
Diaphyseal tibia	22 (29.7%)
Distal metaphyseal tibia	29 (39.2%)
**NUSS**	
Average (SD)—points	69.9 ± 8.6
Patients > 75 points—no (%)	24 (32.4%)
**Previous surgical procedures—no**	
Median (range)	3 (1–32)
**Time since the injury—months**	
Median (range)	14.3 (0.7–298.1)
**Candidates for amputation before our intervention—no (%)**	33 (46.5%)

SD: standard deviation; IR: Interquartile range; NUSS: Non-Union Scoring System.

**Table 2:** Treatment description.

**Main Surgical Procedure**	
**Variable**	**Value**
**Removed hardware—no (%)**	
No hardware	27 (36.48%)
Plates and screws	17 (22.97%)
External fixators	13 (17.56%)
Intramedullary nails	12 (16.21%)
Other (screws, cement spacer)	4 (5.4%)
**Treatment strategy after resection**	
One-stage ASR	32 (43.24%)
Two-stage ASR	10 (13.51%)
One-stage BT (without DS revision)	14 (18.91%)
Two-stage BT (with DS revision)	10 (13.51%)
Fixation	8 (10.81%)
**Bone defect—cm**	
Average ± SD	5.4 ± 2.7
**Surgical time—minutes**	
Average ± SD	196.1 ± 43.4
**Time from 1st to 2nd surgical stage—months**	
Average ± SD	4.7 ± 3.1
**Hospital stay—days**	
Median (range)	4 (1–21)
**Need for blood transfusion—no (%)**	
	1 (1.35%)
**Culture exam results—no (%)**	
Monomicrobial	38 (51.35%)
Polymicrobic	15 (20.27%)
Negative	17 (22.97%)
N/A	4 (5.4%)
**Isolated microorganisms—no (%)**	
Staphylococcus aureus	24%
Other staphylococci (epidermidis, capitis, haemolyticus)	32%
Enterobacteriaceae (E. cloacae, K. pneumoniae, E. coli)	15%
Enterococci (faecalis, faecium)	12%
Pseudomonas aeruginosa	6%
Others (P. mirabilis, A. niger, S. mitis, C. striatum)	8%
**Unplanned Revisions or Re-Interventions**	
**Variable**	**Value**
**UR—no (%)**	
None	63 (85.13%)
One	10 (13.51%)
Two *	3 (4.05%)
**Time to 1st UR—months**	
Average ± SD	7.44 ± 2.11
**Reason for UR—no (%)**	
Delayed union	5
Half-pin/wires loosening	3
Infection persistence	2
**Re-interventions—no (%)**	11 (14.86%)
Re-fractures	10 (13.51%)
Infection recurrence	1 (1.35%)

ASR: acute shortening and re-lengthening; BT: bone transport; UR: unplanned revision; DS: docking site; SD: standard deviation. * for correction of residual deformity or early consolidation.

**Table 3:** Outcomes and complications.

Outcomes	
Clinical Outcome	Value
**EFT—months**	
Average ± SD	11.5 ± 4.4
**EFI—months/cm**	
Average ± SD	2.49 ± 1.09
**Bone union rate—no (%)**	
	73 (98.65%)
**Infection healing rate—no (%)**	
	68 (91.89%)
**ASAMI Bone—no (%)**	
Excellent	63 (85.14%)
Good	9 (12.16%)
Fair	1 (1.35%)
Poor	1 (1.35%)
**ASAMI Functional—no (%)**	
Excellent	38 (51.35%)
Good	28 (37.84%)
Fair	7 (9.46%)
Poor	0
Failure	1 (1.35%)
**Walking aids—%**	
No need	92%
Crutch for long distances	23%
Wheelchair for long distances	4%
**Returned to work—%**	
Total	93%
Same task as before injury	77%
Re-assigned	16%
**PGIC**	
Very much improved	89%
Much improved	7.8%
Minimally improved	1.5%
No change	0
Minimally worse	1.5%
Much worse	0
Very much worse	0

EFT: external-fixation time; EFI: external-fixation index; SD: standard deviation; PGIC: patient global impression of change; UR: unplanned revision.

## Data Availability

The original data presented in the study are openly available in https://docs.google.com/spreadsheets/d/1cOpzxE2BEUVYeOMiQm1Mc8A3qFbYb2zr/edit?usp=sharing&ouid=108213801648630774753&rtpof=true&sd=true (accessed on 16 September 2024).
